# Supplementary material for: Effect of voicing and articulation manner on aerosol particle emission during human speech
Source: PLoS One. 2020 Jan 27;15(1):e0227699. doi: 10.1371/journal.pone.0227699 (PMC6984704; doi:10.1371/journal.pone.0227699)
Supplement: S1 Table — Blue and green columns show the number and fraction of each phoneme category in phrase, respectively. (PDF) [file pone.0227699.s001.pdf]

S1 Table. Details of phonetic analysis for Rainbow passage. Blue and green columns show the number and fraction of each phoneme category in phrase, respectively.

Phrase Number	Phrases	Syllables Number	IPA Phonation	Vowels		Voiced Plosives		Voiceless Plosives		Voiced Fricatives		Voiceless Fricatives		Nasals		Approx.		Trills	
1	When the sunlight strikes raindrops in the air,	10	wɛn ðə ˈsʌnˌlaɪt straɪks ˈreɪnˌdrɑps ɪn ði ɛr,	13	0.382	1	0.029	4	0.118	2	0.059	4	0.118	4	0.118	2	0.059	4	0.118
2	they act as a prism and form a rainbow.	10	ðeɪ ækt æz ə ˈprɪzəm ænd fɔrm ə ˈreɪnˌboʊ.	14	0.467	1	0.033	3	0.1	4	0.133	1	0.033	4	0.133	0	0	3	0.1
3	The rainbow is a division of white	10	ðə ˈreɪnˌboʊ ɪz ə dɪˈvɪʒən ʌv waɪt	13	0.52	2	0.08	1	0.04	5	0.2	0	0	2	0.08	1	0.04	1	0.04
4	light into many beautiful colors.	11	laɪt ˈɪntu ˈmɛni ˈbjutəfəl ˈkʌlərz.	11	0.423	1	0.038	4	0.154	1	0.038	1	0.038	3	0.115	4	0.154	1	0.038
5	These take the shape of a long round arch,	9	ðiz teɪk ðə ʃeɪp ʌv ə lɔŋ raʊnd ɑrʧ,	12	0.429	1	0.036	4	0.143	4	0.143	2	0.071	2	0.071	1	0.036	2	0.071
6	with its path high above, and its two ends	10	wɪð ɪts pæθ haɪ əˈbʌv, ænd ɪts tu ɛndz	11	0.393	2	0.071	4	0.143	4	0.143	4	0.143	2	0.071	1	0.036	0	0
7	apparently beyond the horizon.	10	əˈpɛrəntli bɪˈɑnd ðə həˈraɪzən.	11	0.458	2	0.083	2	0.083	2	0.083	1	0.042	3	0.125	1	0.042	2	0.083
8	There is, according to legend,	8	ðɛrɪz, əˈkɔrdɪŋ tu ˈlɛʤənd,	8	0.381	3	0.143	2	0.095	3	0.143	0	0	2	0.095	1	0.048	2	0.095
9	a boiling pot of gold at one end.	9	ə ˈbɔɪlɪŋ pɑt ʌv goʊld æt wʌn ɛnd.	10	0.417	4	0.167	3	0.125	1	0.042	0	0	3	0.125	3	0.125	0	0
10	People look but no one ever finds it.	10	ˈpipəl lʊk, bʌt noʊ wʌn ˈɛvər faɪndz ɪt.	12	0.414	3	0.103	4	0.138	2	0.069	1	0.034	3	0.103	3	0.103	1	0.034
11	When a man looks for something beyond	10	wɛn ə mən lʊks fɔr ˈsʌmθɪŋ bɪˈɑnd	9	0.36	2	0.08	1	0.04	0	0	4	0.16	6	0.24	2	0.08	1	0.04
12	his reach, his friends say he is looking for	10	hɪz riʧ, hɪz frɛndz seɪ hi ɪz ˈlʊkɪŋ fɔr	11	0.355	1	0.032	2	0.065	4	0.129	7	0.226	2	0.065	1	0.032	3	0.097
13	the pot of gold at the end of the rainbow.	11	ðə pɑt ʌv goʊld æt ði ɛnd ʌv ðə ˈreɪnˌboʊ.	14	0.467	4	0.133	3	0.1	3	0.1	2	0.067	2	0.067	1	0.033	1	0.033
14	Throughout the centuries people have	9	θruˈaʊt ðə ˈsɛnʧəriz ˈpipəl hæv	10	0.4	0	0	4	0.16	3	0.12	4	0.16	1	0.04	1	0.04	2	0.08
15	explained the rainbow in various ways.	10	ɪkˈspleɪnd ðə ˈreɪnˌboʊ ɪn ˈvɛriəs weɪz.	14	0.467	2	0.067	2	0.067	3	0.1	2	0.067	3	0.1	2	0.067	2	0.067
16	Some have accepted it as a miracle	10	sʌm hæv ækˈsɛptɪd ɪt æz ə ˈmɪrəkəl	10	0.4	1	0.04	5	0.2	2	0.08	3	0.12	2	0.08	1	0.04	1	0.04
17	without physical explanation.	9	wɪˈθaʊt ˈfɪzɪkəl ˌɛkspləˈneɪʃən.	11	0.44	0	0	4	0.16	2	0.08	3	0.12	2	0.08	3	0.12	0	0
18	To the Hebrews it was a token that	10	tu ðə ˈhibruz ɪt wʌz ə ˈtoʊkən ðæt	12	0.444	2	0.074	5	0.185	4	0.148	1	0.037	1	0.037	1	0.037	1	0.037
19	there would be no more universal floods.	10	ðɛr wʊd bi noʊ mɔr ˌjunəˈvɜrsəl flʌdz.	10	0.357	3	0.107	0	0	3	0.107	2	0.071	3	0.107	4	0.143	3	0.107
20	The Greeks used to imagine that it was	10	ðə griks juzd tu ɪˈmæʤən ðæt ɪt wʌz	10	0.357	3	0.107	4	0.143	5	0.179	1	0.036	2	0.071	2	0.071	1	0.036
21	a sign from the gods to foretell war	10	ə saɪn frʌm ðə gɑdz tu fɔrˈtɛl wɔr	10	0.385	2	0.077	2	0.077	2	0.077	3	0.115	2	0.077	2	0.077	3	0.115
22	or heavy rain. The Norsemen considered	11	ɔr ˈhɛvi reɪn. ðə ˈnɔrsmɪn kənˈsɪdərd	11	0.393	2	0.071	2	0.071	2	0.071	2	0.071	5	0.179	0	0	4	0.143
23	the rainbow as a bridge over which the gods	10	ðə ˈreɪnˌboʊ æz ə brɪʤ ˈoʊvər wɪʧ ðə gɑdz	14	0.438	5	0.156	1	0.031	6	0.188	1	0.031	1	0.031	1	0.031	3	0.094
24	passed from earth to their home in the sky.	9	pæst frʌm ɜrθ tu ðɛr hoʊm ɪn ðə skaɪ.	11	0.393	1	0.036	3	0.107	2	0.071	5	0.179	3	0.107	0	0	3	0.107
25	Others have tried to explain the	8	ˈʌðərz hæv traɪd tu ɪkˈspleɪn ðə	10	0.4	1	0.04	4	0.16	4	0.16	2	0.08	1	0.04	1	0.04	2	0.08
26	phenomenon physically.	8	fəˈnɑməˌnɑn ˈfɪzɪkəli.	8	0.471	0	0	1	0.059	1	0.059	2	0.118	4	0.235	1	0.059	0	0
27	Aristotle thought that the rainbow was caused	11	ˈɛrəˌstɑtəl θɔt ðæt ðə ˈreɪnˌboʊ wʌz kɑzd	13	0.419	2	0.065	5	0.161	4	0.129	2	0.065	1	0.032	2	0.065	2	0.065
28	by reflection of the sun's rays by the rain.	11	baɪ rəˈflɛkʃən ʌv ðə sʌnz reɪz baɪ ðə reɪn.	15	0.455	2	0.061	1	0.03	5	0.152	3	0.091	3	0.091	1	0.03	3	0.091
29	Since then physicists have found that it is not	11	sɪns ðɛn ˈfɪzɪsɪsts hæv faʊnd ðæt ɪt ɪz nɑt	12	0.353	1	0.029	4	0.118	5	0.147	8	0.235	4	0.118	0	0	0	0
30	reflection, but refraction by the raindrops	11	rəˈflɛkʃən, bʌt ˌriˈfrækʃən baɪ ðə ˈreɪnˌdrɑps	13	0.371	3	0.086	4	0.114	1	0.029	5	0.143	3	0.086	1	0.029	5	0.143
31	which causes the rainbows. Many complicated	10	wɪʧ ˈkɑzəz ðə ˈreɪnˌboʊz. ˈmɛni ˈkɑmpləˌkeɪtəd	15	0.429	2	0.057	6	0.171	4	0.114	1	0.029	4	0.114	2	0.057	1	0.029
32	ideas about the rainbow have been formed.	10	aɪˈdiəz əˈbaʊt ðə ˈreɪnˌboʊ hæv bɪn fɔrmd.	15	0.484	5	0.161	1	0.032	3	0.097	2	0.065	3	0.097	0	0	2	0.065
33	The difference in the rainbow depends	10	ðə ˈdɪfərəns ɪn ðə ˈreɪnˌboʊ dɪˈpɛndz	11	0.407	4	0.148	1	0.037	3	0.111	2	0.074	4	0.148	0	0	2	0.074
34	considerably upon the size of	10	kənˈsɪdərəbli əˈpɑn ðə saɪz ʌv	11	0.458	2	0.083	2	0.083	3	0.125	2	0.083	2	0.083	1	0.042	1	0.042
35	the drops and the width of the colored band	10	ðə drɑps, ænd ðə wɪdθ ʌv ðə ˈkʌlərd bænd	10	0.333	6	0.2	2	0.067	4	0.133	2	0.067	2	0.067	2	0.067	2	0.067
36	increases as the size of the drops increases.	10	ɪnˈkrisəz æz ðə saɪz ʌv ðə drɑps ɪnˈkrisəz.	13	0.394	1	0.03	3	0.091	7	0.212	4	0.121	2	0.061	0	0	3	0.091
37	The actual primary rainbow observed	10	ði ˈækʧuəl ˈpraɪˌmɛri ˈreɪnˌboʊ əbˈzɜrvd	14	0.452	3	0.097	3	0.097	3	0.097	1	0.032	2	0.065	1	0.032	4	0.129
38	is said to be the effect of super-	10	ɪz sɛd tu bi ði ɪˈfɛkt ʌv ˈsupər	10	0.435	2	0.087	4	0.174	3	0.13	3	0.13	0	0	0	0	1	0.043
39	imposition of a number of bows.	10	ɪmpəˈzɪʃən ʌv ə ˈnʌmbər ʌv baʊz.	11	0.458	2	0.083	1	0.042	4	0.167	1	0.042	4	0.167	0	0	1	0.042
40	If The red of The second bow falls upon	11	ɪf ðə rɛd ʌv ðə ˈsɛkənd baʊ fɔlz əˈpɑn	12	0.429	3	0.107	2	0.071	4	0.143	3	0.107	2	0.071	1	0.036	1	0.036
41	the green of the first the result is to give	11	ðə grin ʌv ðə fɜrst, ðə rɪˈzʌlt ɪz tu gɪv	11	0.367	2	0.067	3	0.1	7	0.233	2	0.067	1	0.033	1	0.033	3	0.1
42	a bow with an abnormally wide yellow band,	12	ə baʊ wɪð ən æbˈnɔrməli waɪd ˈjɛloʊ bænd,	15	0.484	5	0.161	0	0	1	0.032	0	0	4	0.129	5	0.161	1	0.032
43	since red and green light when mixed form yellow.	10	sɪns rɛd ænd grin laɪtwɛn mɪkst fɔrm ˈjɛloʊ.	12	0.343	3	0.086	3	0.086	0	0	4	0.114	6	0.171	4	0.114	3	0.086
44	This is a very common type of bow,	10	ðɪs ɪz ə ˈvɛri ˈkɑmən taɪp ʌv baʊ,	12	0.5	1	0.042	3	0.125	4	0.167	1	0.042	2	0.083	0	0	1	0.042
45	one showing mainly red and yellow,	9	wʌn ˈʃoʊɪŋ ˈmeɪnli rɛd ænd ˈjɛloʊ,	12	0.48	2	0.08	0	0	0	0	1	0.04	5	0.2	4	0.16	1	0.04
46	with little or no green or blue.	8	wɪð ˈlɪtəl ɔr noʊ grin ɔr blu.	8	0	2	0.095	1	0.048	1	0.048	0	0	2	0.095	4	0.19	3	0.143
