# Supplementary material for: Effect of voicing and articulation manner on aerosol particle emission during human speech
Source: PLoS One. 2020 Jan 27;15(1):e0227699. doi: 10.1371/journal.pone.0227699 (PMC6984704; doi:10.1371/journal.pone.0227699)
Supplement: S1 Fig — Particle emission rate (NV)/concentration (CV) versus root mean square amplitude, Arms, while saying (A) /ɑ/ (the vowel sound in ‘saw’), (B) /i/ (the vowel sound in ‘need’), and (C) /u/ (the vowel sound in ‘mood’) for 4 different amplitudes by 10 participants, 6 males (denoted as M1 to M6), and 4 females (denoted as F1 to F4). Solid lines are power law fits with exponent (A) 0.80, (B) 0.70, and (C) 0.91, correlation coefficient (A) 0.54, (B) 0.14, and (C) 0.34, and Pearson’s p value (A) 2.8×10^−4^, (B) 0.37, and (C) 0.03. (PDF) [file pone.0227699.s002.pdf]

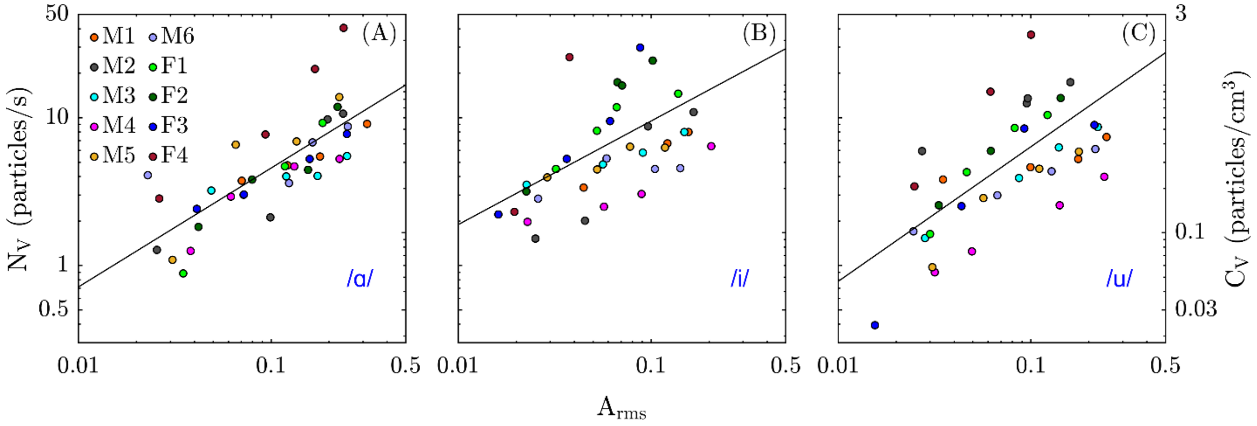

S1 Fig. Particle emission rate/concentration of vowels. Particle emission rate (N_V)/concentration (C_V) versus root mean square amplitude, A_{rms}, while saying (A) /ɑ/ (the vowel sound in 'saw'), (B) /i/ (the vowel sound in 'need'), and (C) /u/ (the vowel sound in 'mood') for 4 different amplitudes by 10 participants, 6 males (denoted as M1 to M6), and 4 females (denoted as F1 to F4). Solid lines are power law fits with exponent (A) 0.80, (B) 0.70, and (C) 0.91, correlation coefficient (A) 0.54, (B) 0.14, and (C) 0.34, and Pearson's p value (A) 2.8×10^{-4}, (B) 0.37, and (C) 0.03.
