# Supplementary material for: Effect of voicing and articulation manner on aerosol particle emission during human speech
Source: PLoS One. 2020 Jan 27;15(1):e0227699. doi: 10.1371/journal.pone.0227699 (PMC6984704; doi:10.1371/journal.pone.0227699)
Supplement: S2 Fig — Boxplot of particle emission rate (NM)/concentration (CM) while repeating 12 monosyllabic words, NM, (sample size n = 10). Top x-axis shows the IPA notation of each word. (PDF) [file pone.0227699.s003.pdf]

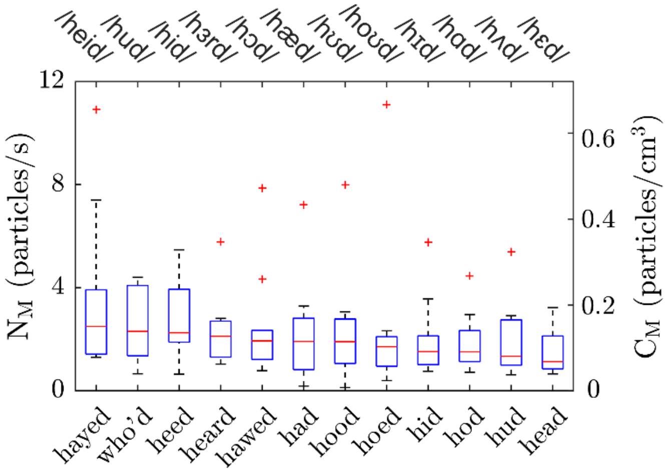

S2 Fig. Particle emission rate/concentration of monosyllabic words. Boxplot of particle emission rate (N_M)/concentration (C_M) while repeating 12 monosyllabic words, N_M, (sample size n = 10). Top x-axis shows the IPA notation of each word.
