# Supplementary material for: Effect of voicing and articulation manner on aerosol particle emission during human speech
Source: PLoS One. 2020 Jan 27;15(1):e0227699. doi: 10.1371/journal.pone.0227699 (PMC6984704; doi:10.1371/journal.pone.0227699)
Supplement: S3 Fig — Boxplot of particle emission rate (ND)/concentration (CD) while repeating 14 disyllabic words, ND, (sample size n = 30). Each background color represents the words including consonants which have similar voicing and articulation manner. (PDF) [file pone.0227699.s004.pdf]

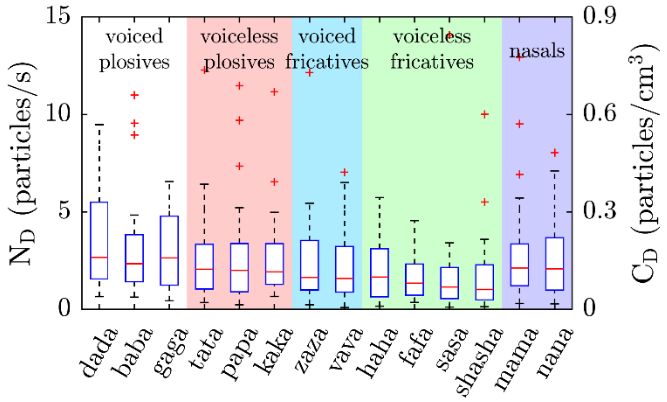

S3 Fig. Particle emission rate/concentration of disyllabic words. Boxplot of particle emission rate (N_D)/concentration (C_D) while repeating 14 disyllabic words, N_D, (sample size n = 30). Each background color represents the words including consonants which have similar voicing and articulation manner.
