# Supplementary material for: Effect of voicing and articulation manner on aerosol particle emission during human speech
Source: PLoS One. 2020 Jan 27;15(1):e0227699. doi: 10.1371/journal.pone.0227699 (PMC6984704; doi:10.1371/journal.pone.0227699)
Supplement: S4 Fig — Bar plots of particle emission rate (NR)/concentration (CR) versus time for reading Rainbow passage for 18 participants (10 males denoted as M7 to M16, and 8 females denoted as F5 to F12). (PDF) [file pone.0227699.s005.pdf]

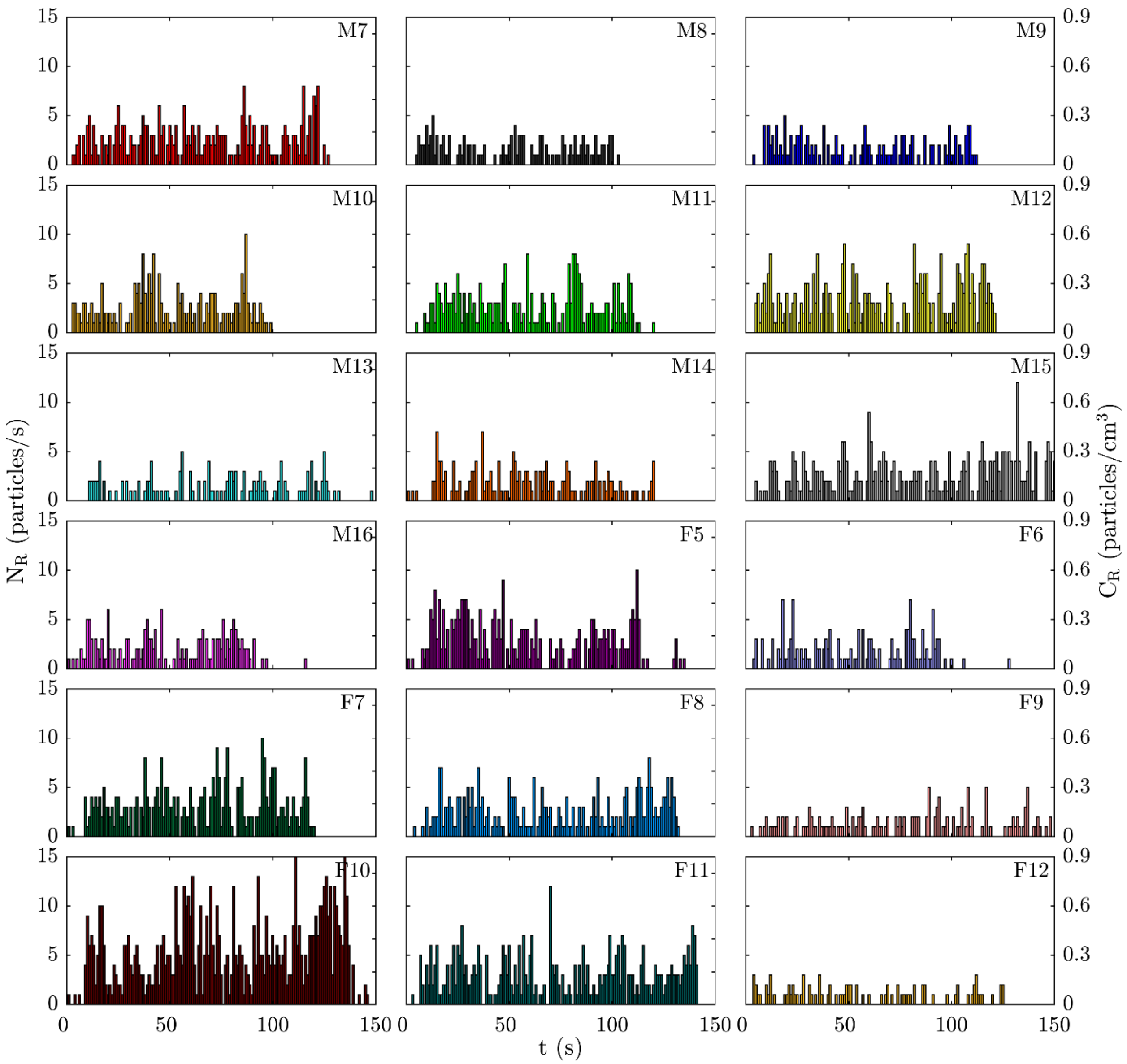

S4 Fig. Particle emission rate/concentration for reading Rainbow passage. Bar plots of particle emission rate/concentration versus time for reading Rainbow passage for 18 participants (10 males denoted as M7 to M16, and 8 females denoted as F5 to F12).
