# Supplementary material for: Effect of voicing and articulation manner on aerosol particle emission during human speech
Source: PLoS One. 2020 Jan 27;15(1):e0227699. doi: 10.1371/journal.pone.0227699 (PMC6984704; doi:10.1371/journal.pone.0227699)
Supplement: Attachment — Submitted filename: asadi_PLoS_ONE_referee_response.pdf [file pone.0227699.s006.pdf]

Response to Reviewer #1 for paper #PONE-D-19-20208, "Effect of Voicing and Articulation Manner on Aerosol Particle Emission during Human Speech" submitted to PLOS ONE by Asadi et al.

We thank the Referee for their comments and detailed assessments, which have helped us to clarify and improve the manuscript. Detailed responses are below.

> *This manuscript reports a new set of measurements of the roles of "phones" in expired droplet generation following their early work.*
>
> Asadi S, Wexler AS, Cappa CD, Barreda S, Bouvier NM, Ristenpart WD. Aerosol emission and superemission during human speech increase with voice loudness. Scientific Reports. 2019;9:2348.
>
> The authors now found that the "certain phones are associated with significantly higher particle production", which is not surprising following the existing data in the literature, but with a more detailed exploration.

We thank the reviewer for their assessment that the results presented here are "not surprising," but we admit that the basis for this assessment is unclear to us. To our knowledge, our study is the first to show that specific phones yield significantly more expiratory aerosols than others during human speech. If the referee is aware of prior experimental work demonstrating this behavior, we will be happy to add the citations to our manuscript.

> A total of 56 subjects were tested in total, however, for each group of tests, e.g. vowel experiments, only 14 subjects were tested. Except the disyllabic word experiments, the sample size is not large, which is a pity.

We agree that sample size is an important consideration, which hinges on what "large" means in terms of statistical significance. Please see our response to reviewer 3 for a more detailed discussion of our sample size statistical calculations.

> It is important to recognize that the authors only measured the droplet sizes at the site of the measurement, i.e. somewhere within APS, not at the exit. On the journey from the mouth exit, entering the funnel, flowing through the connection tube, the droplets are expected to evaporate and different sizes of droplets would experience different evaporation rates. This should probably be added in the limitation discussion.

We thank the reviewer for making this point. We completely agree that the evaporation time scales for micron-sized droplets (on the order of 100 ms) are invariably small compared to their travel time to the APS measurement module (approximately 3 seconds in our setup). Therefore the particles measured here had fully dried into droplet nuclei prior to measurement. We provided more detailed explanations of these time scales in our previous work (Asadi et al., *Scientific Reports*, 9, 2348, 2019), where we pointed out that, if desired, different correction factors available in the literature can be used to estimate the initial diameter of the particles (e.g., Nicas et al., *Journal of Occupational and Environmental Hygiene,* 2, 143–154, 2005). In the context of airborne disease transmission, however, please note that what matters the most is the size of the droplets upon

inhalation by a susceptible individual, i.e., in their fully dried state, as reported here. To clarify this point, we have added text to page 5 of the revised manuscript.

> Only a normalized particle emission rate is presented to address significant individual emission differences. It is not surprising to me that different phones produce different number of droplets. We are more interested how many are produced. The authors should provide the exact emission rates n some suitable format at least in Supplementary Information. I could not access S1 so that the information might be there (but sounds only for Rainbow passage).

We appreciate the reviewer raising this issue. As requested we have added three supplementary figures (S1 through S3) to the supporting information to provide the raw emission rates for the "vowels", "monosyllabic words", and "disyllabic words" experiments. The main text has also been revised to direct interested readers to these figures.

> The authors discussed their work in terms of the speculation by Inouye (2003) (note not an original paper, and no data was presented) and Inouye and Sugihara (2015) where some kinds of pressure was measured. Both are not a great paper. In Inouye and Sugihara (2015), the claim that Japanese language seems to produce a smaller pressure than English and Chinese. Many questions existing in their measurement if one reads their paper carefully. I have personally experienced when some Japanese friends spoke "wildly" after drink, I did not see any such "low pressure" phenomenon. I believe that the statements made in this para are not appropriate.

The reviewer raises interesting points. Our intent in citing the work by Inouye et al. was not to comment on the quality thereof (which we agree is debatable); rather, we are unaware of any other published scientific work that broached the hypothesis that the phonetic characteristics of human speech could modulate the rate of airborne disease transmission. Since our experimental measurements indicate that different phones do release different quantities of expiratory particles, and since this result has implications for airborne disease transmission, we believe it is best to give credit where credit is due and to cite the group that first introduced the hypothesis.

Although we have no reason to doubt the referee's personal observations of their Japanese friends, we prefer to restrict our scientific discussion to work that is published and available to all. Toward that end, we believe our description of the work of Inouye accurately summarizes both their initial speculative hypothesis and their subsequent investigation of eggressive flow rates.

> The work by the authors in this manuscript in Asadi et al (2019) showed that there were no statistically significant difference in overall aerosol particle emissions for English, Spanish, Mandarin and Arabic, though "distribution of phones in the translated texts was neither measured nor controlled". Why not measure it as you have the original text?

We agree that since we have the original texts, it is possible in principle to analyze the precise distribution of phones in text passages read aloud in the languages tested in our previous work

(Spanish, Mandarin, Arabic). The main difficulty is that none of us (the co-authors) are fluent in any of those languages, so we focused on performing more controlled experiments that do not depend on language spoken (as reported in this manuscript). We have modified the conclusions of the revised text to note this point and that the raw data is available for interested researchers.

Response to Reviewer #2 for paper #PONE-D-19-20208, "Effect of Voicing and Articulation Manner on Aerosol Particle Emission during Human Speech" submitted to PLOS ONE by Asadi et al.

> *The paper presents measurements of aerosol production during various parts of human speech. The work is unique and interesting, and provides insights into human aerosol production, which is an important factor in airborne disease transmission. The paper is well written and easy to follow, which is greatly appreciated. I have only a few minor comments.*

We thank the Referee for the comments and insightful questions, which have helped us to clarify and improve the manuscript. Detailed responses are below.

> *The authors compare aerosol production during the enunciation of different vowel sounds, and during the enunciation of different consonants. Can they compare the relative aerosol production for vowels vs. consonants? Figure 6A might suggest that vowels produce more aerosols than consonants, but I may be misinterpreting the data. If the authors' data doesn't allow them to make this comparison, that is fine, but if the comparison is possible, then it would be interesting.*

This is an excellent point. The data presented in Fig. 6A suggest that vowels emit more aerosol particles than at least voiceless fricatives. One reason is that vowels are known in general to be louder than consonants and, as we showed in our previous work, louder speech releases more aerosol particles. Furthermore, there is no obstruction in the vocal tract when producing vowels, so there is no barrier to airflow that carries particles out. Finally the laryngeal particle generation mode present for voiced phonemes such as vowels does not contribute to the particle generation rate of voiceless fricatives.

In this study however we were not able to compare particle emission rate for vowels and consonants directly since the disyllabic words used here to compare the consonants also include vowels. We modified the text to explain this point.

> *The normal breathing rate for an adult at rest is typically 5-10 liters/minute. As the authors note, their aerosol spectrometer draws air at 5 liters/minute, so they are measuring a sample of the exhaled breath, not all of the particles that are exhaled. Does the rate of exhalation vary significantly during speech? If so, this might affect the measurements, in that if a person was producing twice as many particles when pronouncing one phone as vs. another but also exhaling twice as fast, the aerosol concentration measured by the APS would be the same. On the other hand, if variations in flow due to speech are small, then the effect would be minimal.*

We agree that in our setup some of the exhaled air can be exhausted around the side of the funnel rather than into the APS. Regardless of the exhalation rate, however, the APS continues to draw in air at 5 L/min, i.e., it continuously samples from the air in the funnel inlet and provides a measurement of the number of particles present. Our data clearly show there are more particles present when participants vocalize one word versus another. The APS samples the volume concentration of air in the immediate entrance to the APS, regardless of whether some of that air ends up in the sheath flow or outside of the funnel. To emphasize this point,

we have modified all of our graphs of the particle emission rate in supplementary information (figures S1 to S4) to include a secondary axis specifying the equivalent concentration in particles/cm^3, and revised the text accordingly.

What was the time resolution for the APS? Did it report cumulative counts for every second, or every 2 seconds, or something else?

We thank the Referee for this comment. The time resolution of APS was set to 1 second and it reported cumulative counts for every second. We have revised the methods section to include this information.

In most places in the text, the authors use the term "voiceless", but sometimes they use "unvoiced". Are these terms equivalent? If so, it may be best to use just one term, since I expect that most of the readers (like me) will not be familiar with the terminology of linguistics.

We thank the Referee for pointing this out. The terms "voiceless" and "unvoiced" are equivalent and they refer to sounds that vocal folds do not vibrate and stay open during phonation. We have revised the manuscript to use one term, "voiceless", throughout the manuscript.

If the authors plan to continue this line of research, it would be very interesting to see if a difference in aerosol emission or emission of pathogens could be detected in, for example, people with colds or influenza while they are speaking vs. just breathing.

We appreciate the reviewer raising this question, which we agree is a fascinating topic for future work. We know of at least one paper indicating that cough aerosols produced by patients with cold symptoms demonstrated a significant increase in the number of aerosols during illness compared to when patients were recovered (Lee et al., *Aerosol and Air Quality Research*, 19: 840–853, 2019). A fascinating hypothesis is that a similar increase will be observed for ill patients when talking and breathing compared to healthy subjects. We have added this point and citation to the conclusions to note that it is an important avenue of future research.

Response to Reviewer #3 for paper #PONE-D-19-20208, "Effect of Voicing and Articulation Manner on Aerosol Particle Emission during Human Speech" submitted to PLOS ONE by Asadi et al.

The study aimed (n=56) to investigate the effect of different 'phones' (the basic sound units of speech) on the emission of particles from the human respiratory tract during speech. Certain phones were associated with significantly higher particle production. In phrases, a positive correlation was observed between the vowel content and particle production.

Minor revisions:

Indicate the underlying covariance structure used in the mixed effects linear model and the criteria for selecting it.

The underlying covariance structure used here is the default structure in Stata/IC 15, i.e., "identity," which is short for "multiple of identity". In this structure all variances are assumed equal and all covariances are assumed to be zero. Because we had only one random effect parameter (the between-person variability), this covariance structure is most reasonable for the analysis. We have revised the statistical analysis section to clarify the covariance structure used.

State and justify the study's target sample size with a pre-study statistical power calculation. The power calculation should include: sample size, alpha level (indicating one or two-sided), minimal detectable difference and statistical testing method.

We thank the referee for raising this question. A crucial point to note is that, prior to this work, there were no measurements of aerosol production versus different phones available anywhere in the literature. Our study is the first to investigate this question and was thus exploratory in nature. In other words, we had no data available to inform a pre-study statistical power calculation.

Nonetheless, we can perform a sample post hoc power calculation to compare one pair of our disyllabic word data (e.g., dada vs. papa) to assess statistical power. Stata/IC 15 was used to perform a paired test comparing two correlated means using a 5%-level two-sided test and sample size of n = 30, resulting in a statistical power of 0.89. Similar results are obtained from pairwise comparison of other disyllabic pairs (n = 30) and monosyllabic pairs (n = 10). More complicated power calculations for multi-level mixed effects models can be used; however, in this study we are more interested in establishing the existence of difference between particle emission rates of different phonemes rather than looking for clinically meaningful differences. Our results here serve as a proof of concept to motivate future more detailed work. We have added discussion of these to the main text.
